# Role of the Polarity Protein Scribble for Podocyte Differentiation and Maintenance

**DOI:** 10.1371/journal.pone.0036705

**Published:** 2012-05-07

**Authors:** Björn Hartleben, Eugen Widmeier, Nicola Wanner, Miriam Schmidts, Sung Tae Kim, Lisa Schneider, Britta Mayer, Dontscho Kerjaschki, Jeffrey H. Miner, Gerd Walz, Tobias B. Huber

**Affiliations:** 1 Renal Division, University Hospital Freiburg, Freiburg, Germany; 2 Spemann Graduate School of Biology and Medicine, Albert-Ludwigs-University Freiburg, Freiburg, Germany; 3 Renal Division, Washington University School of Medicine, St. Louis, Missouri, United States of America; 4 Clinical Institute of Pathology, Medical University of Vienna, Vienna, Austria; 5 BIOSS Centre for Biological Signalling Studies, Albert-Ludwigs-University Freiburg, Freiburg, Germany; INSERM, France

## Abstract

The kidney filter represents a unique assembly of podocyte epithelial cells that tightly enwrap the glomerular capillaries with their complex foot process network. While deficiency of the polarity proteins Crumbs and aPKC result in impaired podocyte foot process architecture, the function of basolateral polarity proteins for podocyte differentiation and maintenance remained unclear. Here we report, that Scribble is expressed in developing podocytes, where it translocates from the lateral aspects of immature podocytes to the basal cell membrane and foot processes of mature podocytes. Immunogold electron microscopy reveals membrane associated localisation of Scribble predominantly at the basolateral site of foot processes. To further study the role of Scribble for podocyte differentiation *Scribble^flox/flox^* mice were generated by introducing loxP-sites into the Scribble introns 1 and 8 and these mice were crossed to *NPHS2.Cre* mice and Cre deleter mice. Podocyte-specific *Scribble* knockout mice develop normally and display no histological, ultrastructural or clinical abnormalities up to 12 months of age. In addition, no increased susceptibility to glomerular stress could be detected in these mice. In contrast, constitutive *Scribble* knockout animals die during embryonic development indicating the fundamental importance of Scribble for embryogenesis. Like in podocyte-specific *Scribble* knockout mice, the development of podocyte foot processes and the slit diaphragm was unaffected in kidney cultures from constitutive *Scribble* knockout animals. In summary these results indicate that basolateral polarity signaling via Scribble is dispensable for podocyte function, highlighting the unique feature of podocyte development with its significant apical membrane expansions being dominated by apical polarity complexes rather than by basolateral polarity signaling.

## Introduction

The glomerular filtration barrier is a unique structure characterized by a precise three dimensional framework of podocytes that elaborate long, regularly spaced, interdigitating foot processes, enveloping the glomerular capillaries. Neighbouring podocytes are connected by the slit diaphragm, a specialized cell junction and the only cell-cell contact of mature podocytes, that bridges the filtration slit between podocyte foot processes [1]. We recently demonstrated that the evolutionarily conserved apical Par3-Par6-aPKC complex (Par complex), a fundamental regulator of apicobasal cell polarity, interacts with the slit diaphragm proteins Nephrin and Neph1 [2]. During podocyte differentiation the Par complex and the cell-cell contacts of immature podocytes migrate from apical towards basal aspects of the podocyte cell membrane, where primary processes and foot processes subsequently develop.

In epithelial cells apicobasal cell polarity is established by the asymmetric distribution of three core polarity complexes, the apical Crumbs complex, consisting of Crumbs, PALS1 and PATJ, the apical Par complex localizing at the tight junctions and the basolateral Scribble complex, comprising the proteins Scribble, Dlg and Lgl [3]. In Zebrafish, morpholino knockdown of the apical polarity protein Crumbs2b causes disorganization of podocyte foot process architecture and loss of slit diaphragms [4]. In addition, podocyte-specific deletion of aPKCiota in mice results in foot process effacement, nephrotic syndrome, progressive glomerulosclerosis and death at 3–4 weeks after birth [5], [6] underlining the critical importance of apical polarity complexes for podocyte differentiation and maintenance. However, the relevance of the basolateral Scribble complex for podocyte function is yet completely unclear.

Scribble is a large cytoplasmic scaffold protein of the leucine-rich repeat (LRR) and PDZ domain (LAP) family with 16 N-terminal LRRs and 4 C-terminal PDZ domains [7]. Targeting of LAP family members to the lateral membrane depends on their LRR domains [8]. In polarized renal epithelial cells Scribble localizes to the adherens junctions and the lateral membrane in an E-Cadherin dependant manner [9], and knockdown of Scribble results in delayed tight junction assembly, increased cell motility and reduced adhesion similar to the phenotype of E-cadherin knockdown [10]. In wound healing assays Scribble is essential for polarization of migrating cells, recruitment of CDC42 and Rac1 to the leading edge and directed migration [11].

In mice, point mutations of *Scribble* (*circletail* and *rumpelstilzchen* mutations) cause severe impairment of neural tube development with craniorachischisis and neonatal death [12], [13]. A similar phenotype is described for the *loop-tail* mouse [14], which displays a mutation in the gene encoding for the planar cell polarity (PCP) protein Vangl2 [15], [16]. Both proteins interact genetically and physically [17], [18], [19].

Scribble is targeted to proteasomal degradation by the high risk papilloma virus protein E6-E6AP ubiquitin-protein ligase complex [20], and cervical neoplasms are associated with reduced Scribble protein levels [21]. Further, downregulation and mislocalization of Scribble promotes cell transformation and mammary tumorigenesis [22], suggesting Scribble as a tumor suppressor. In recently published work *Scribble* heterozygosity causes prostate hyperplasia, while prostate-specific knockout of *Scribble* results in loss of cellular polarity, elevated proliferation and progression to intraepithelial neoplasia [23]. These data underline the fundamental role of Scribble for the establishment and maintenance of epithelial cell polarity, cell migration and tissue architecture.

Here we analysed the spatiotemporal expression of Scribble during glomerular development and generated podocyte-specific and constitutive *Scribble* knockout mice to investigate the role of Scribble in podocyte differentiation and maintenance.

## Results

### Translocation of apical and basolateral polarity proteins during podocyte differentiation

**Figure 1:**
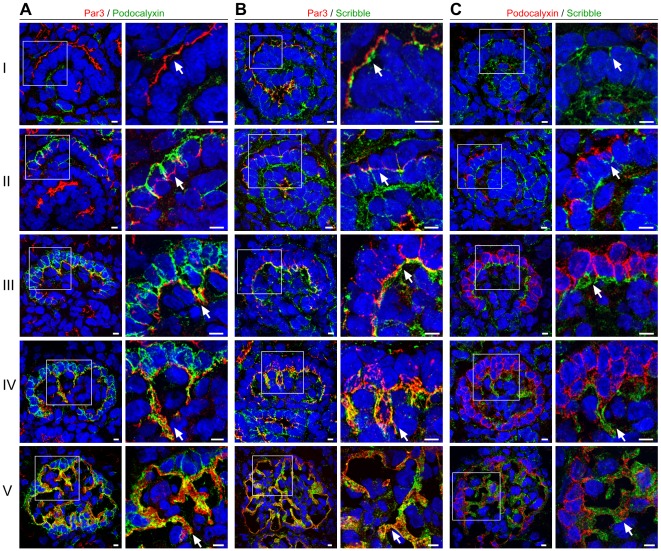
Migration of apical and basolateral polarity proteins during podocyte differentiation. Frozen kidney sections of newborn Wistar rat (P0) were stained using antibodies against the apical membrane protein Podocalyxin, the apical polarity protein Par3 and the basolateral polarity protein Scribble and were subjected to confocal laser microscopy. Since glomerular development is asynchronous, kidneys of newborn rats display various glomerular developmental stages. Each panel displays the expression pattern of the accordant proteins during glomerular development (from left to right): Developmental stages ranging from comma-shaped body (I), s-shaped body (II), capillary loop stage (III to IV), to a maturing glomerulus (V). (**A**) Whereas Par3 is expressed during comma-shaped body stage and localizes to the apical sited cell-cell junctions, expression of Podocalyxin starts during s-shaped body stage, when Par3 and the cell-cell contacts translocate along the lateral side of immature podocytes to basal. During this translocation the apical membrane area, marked by Podocalyxin, increases while the basolateral membrane area shrinks relatively. Arrows indicate translocation of Par3 from the apical cell-cell contacts in I to the developing foot processes in V. (**B**) Scribble localizes basal of Par3 at the cell-cell junctions and at the basolateral membrane during comma-shaped body stage (I) and translocates like Par3 during podocyte differentation to the developing foot processes in V. (**C**) While Podocalyxin and Par3 as well as Par3 and Scribble display an partial overlap of their localization (yellow in A and B), no overlap of Podocalyxin and Scribble can be detected indicating a localization to completely distinct membrane areas with Podocalyxin as an apical membrane marker and Scribble as a basolateral marker protein. Scale bars: 5 µm.

**Figure 2:**
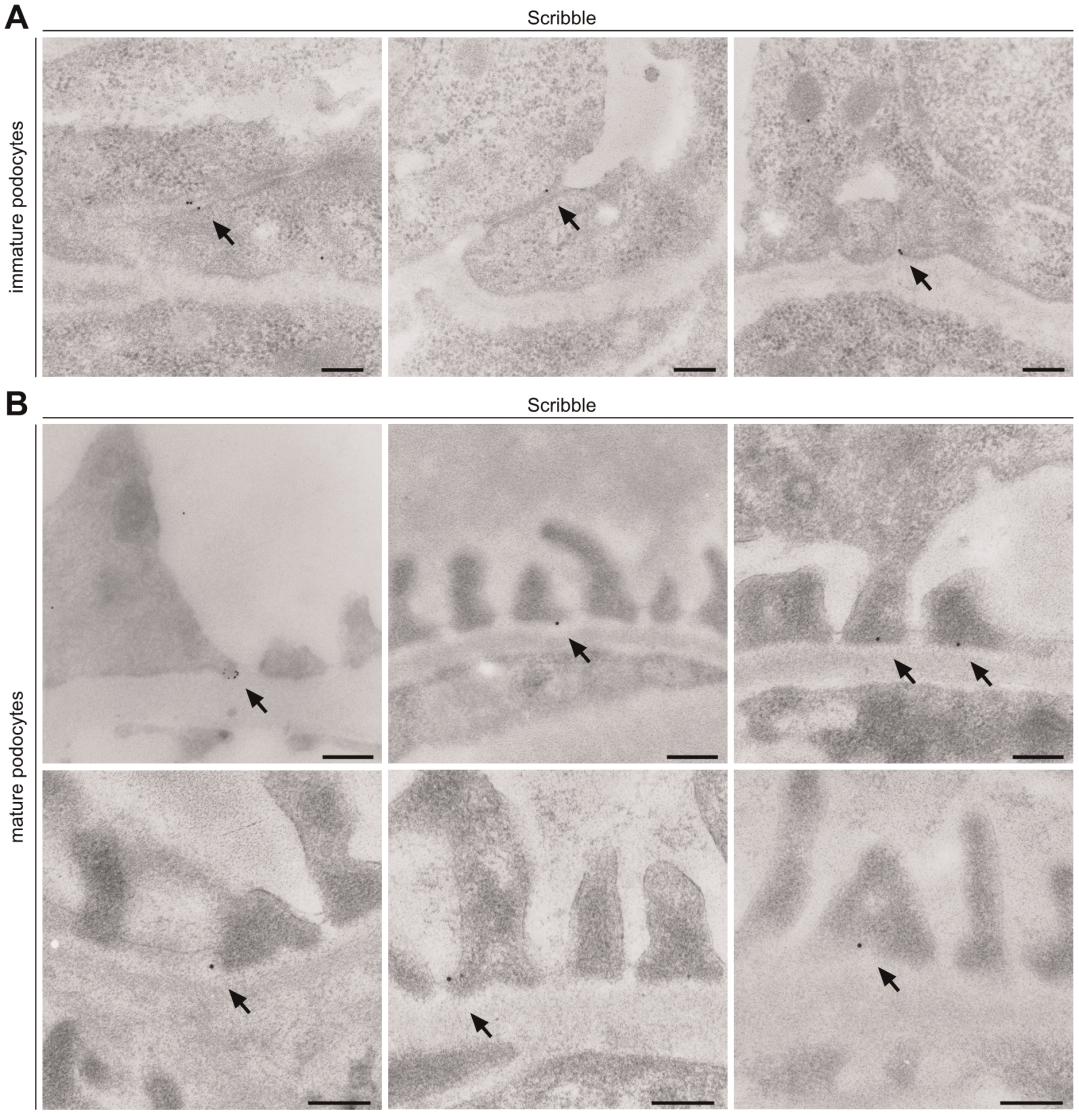
Scribble localizes to the cell-cell contacts and the basolateral membrane of podocytes. (**A**) Immunogold electron microscopy of P0 rat kidney sections displays localization of Scribble (arrows) at immature podocyte cell-cell contacts. (**B**) In adult rat kidney sections Scribble localizes predominantly at the basolateral side of podocyte foot processes and partially at the slit-diaphragm. Scale bars: 200 nm.

**Figure 3:**
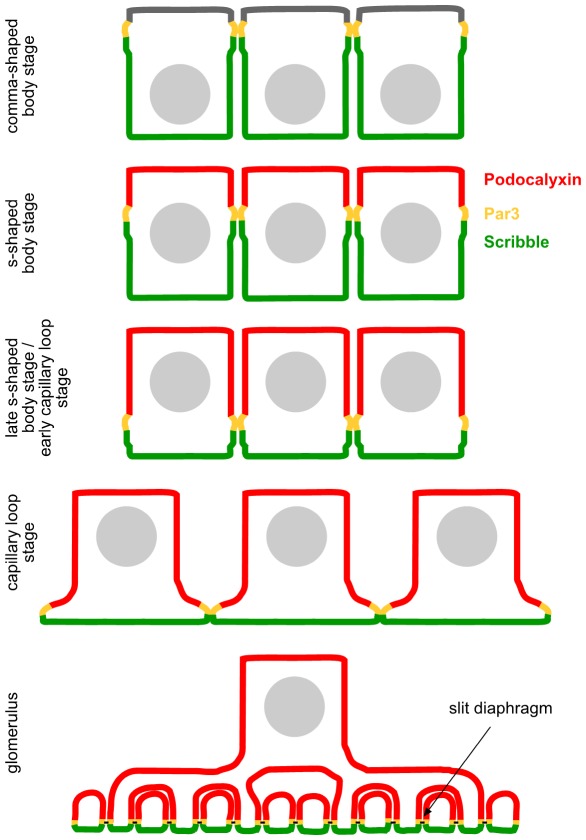
Schematic illustration of the translocation of polarity proteins during podocyte differentiation. During podocyte differentiation the cell-cell contacts translocate from the apical site along the lateral membrane to basal, where primary processes and foot processes develop. Par3 localizes to the cell-cell contacts, moves with them to basal and localizes to the cell-cell contact of mature podocytes, the slit diaphragm, which bridges the filtration slit between foot processes. Scribble localizes basal of Par3 at the cell-cell junctions and the basolateral membrane of immature podocytes. In mature podocytes Scribble localizes to the basolateral membrane, which is defined as the area beneath the slit-diaphragm. Differentiation of podocytes is accompanied with an enlargement of the apical membrane area, marked by Podocalyxin, whose expression starts parallel with the translocation of the cell-cell contacts to basal in s-shaped body stage. Relative to this apical membrane enlargement the basolateral membrane, marked by Scribble, shrinks to the area beneath the slit-diaphragm in mature podocytes.

Previously, we identified that the aPKC complex translocates from the apical to basal membranes during podocyte differentiation, preceding the development of primary and foot processes [5]. To study the protein localisation of Scribble during glomerular development, we co-stained the apical membrane marker Podocalyxin and Par3 with Scribble in newborn rat kidney sections. Whereas Par3 is already expressed during comma-shaped body stage and localizes to the apical sited cell-cell contacts, expression of Podocalyxin starts during s-shaped body stage, when Par3 and the cell-cell contacts migrate along the lateral side of immature podocytes (Figure 1 A). Interestingly, Scribble appears to be enriched basally of Par3 during the comma-shaped body stage and translocates like Par3 during podocyte differentiation to the developing foot processes (Figure 1 B, Figure S1). While Podocalyxin and Par3 as well as Par3 and Scribble display a partial overlap of their localization, no overlap of Podocalyxin and Scribble can be detected indicating localization to completely distinct membrane areas with Podocalyxin being an apical membrane marker and Scribble being a basolateral marker protein (Figure 1 C). To reveal the subcellular localisation of Scribble in podocytes we performed immunogold stainings of newborn and adult rat kidney sections. In immature podocytes Scribble localizes at the cell-cell contacts (Figure 2 A), while in differentiated podocytes it can be detected predominantly at the basolateral side of the foot processes (basal of the slit diaphragm) and also partially at the slit-diaphragm (Figure 2 B). Figure 3 schematically illustrates the localization of Podocalyxin, Par3 and Scribble during podocyte differentiation.

### Generation of podocyte-specific Scribble knockout mice, Scribble^Δpodocyte^

**Figure 4:**
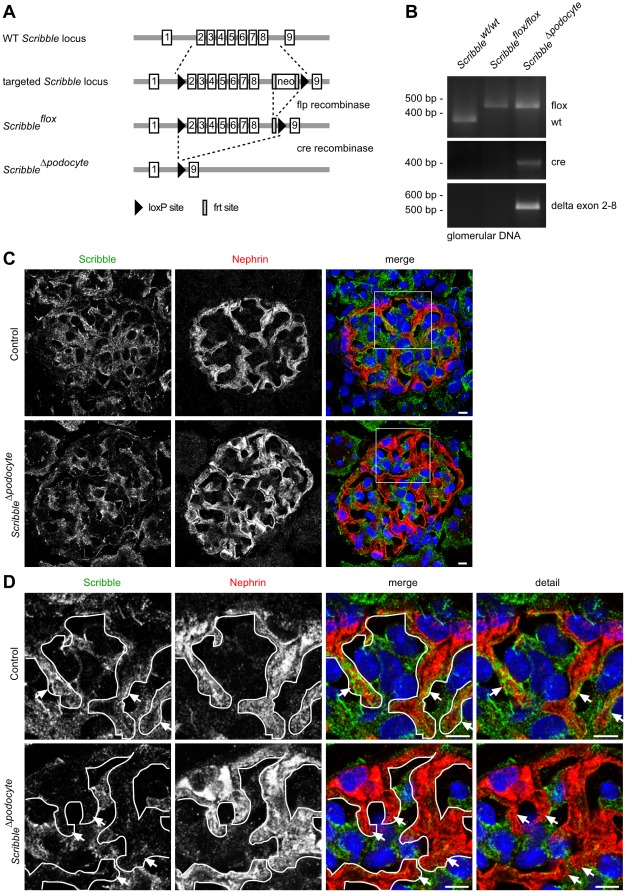
Generation of podocyte-specific Scribble knockout mice, *Scribble^Δpodocyte^*. *Scribble^flox/flox^* mice were crossed with *NPHS2.Cre* mice to generate podocyte-specific *Scribble* knockout mice *Scribble^flox/flox^; NPHS2.Cre* (*Scribble^Δpodocyte^*). (**A**) Generation of tissue-specific *Scribble* knockout mice. (**B**) PCR analysis of genomic DNA from isolated glomeruli confirmed genomic deletion of exon 2–8 in *Scribble^Δpodocyte^* mice. (**C, D**) Frozen kidney sections of control and *Scribble^Δpodocyte^* mice were stained using antibodies against Scribble and the podocyte foot process marker Nephrin and were subjected to confocal laser microscopy. Arrows indicate the podocyte foot process compartment. While Scribble localizes to the podocyte foot process compartment of control mice, no Scribble expression can be detected in podocytes of *Scribble^Δpodocyte^* mice. Scale bars: 5 µm.

To study the role of Scribble for podocyte differentiation *Scribble^flox/flox^* mice were generated by introducing loxP-sites into the introns 1 and 8 flanking *Scribble* exons 2–8 (Figure 4 A). *Scribble^flox/flox^* mice were crossed with *NPHS2.Cre* mice to create podocyte-specific *Scribble* knockout mice (*Scribble^Δpodocyte^*) (Figure 4 B). While Scribble can still be detected in other glomerular cells, co-staining with the podocyte marker protein Nephrin confirmed complete loss of Scribble in podocytes in confocal microscopy (Figure 4 C, D).

### *Scribble^Δpodocyte^* mice develop normal podocyte foot processes and show no increased susceptibility to glomerular stress

**Figure 5:**
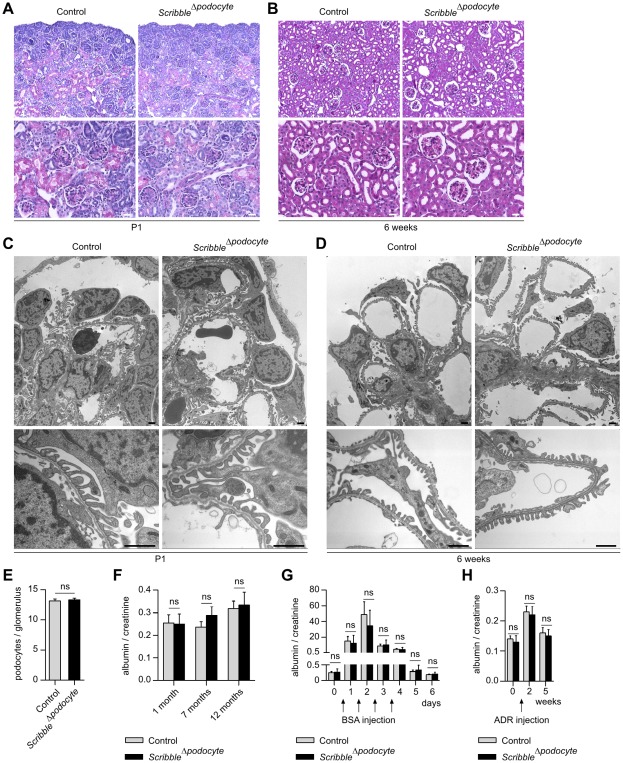
*Scribble^Δpodocyte^* mice develop normal podocyte foot processes and show no increased susceptibility to glomerular stress. (**A, B**) No obvious histological abnormalities can be detected in PAS staining of *Scribble^Δpodocyte^* kidney sections at P1 and at 6 weeks of age compared to control littermates. (**C, D**) Transmission electron micrographs display normal podocyte architecture with foot processes without any obvious ultrastructural defect. (**E**) No difference in the number of podocytes per sectioned glomerulus could be detected (ns, not significant, n = 3 each, 30 glomeruli for each mouse were analyzed). (**F**) During a one year follow up *Scribble^Δpodocyte^* mice develop no significant albuminuria. (**G**) No difference in albuminuria can be detected between *Scribble^Δpodocyte^* mice and control littermates in the BSA-overload model (n = 5 each) and (**H**) the ADR model (n = 6 each). Scale bars: 20 µm in (A) and (B), 1 µm in (C) and (D).

In histological analysis of kidney sections, newborn and adult *Scribble^Δpodocyte^* mice displayed a regular glomerular architecture (Figure 5 A, B), and electron microscopy revealed no abnormalities in podocyte cell body or process morphology (Figure 5 C, D). In addition, no difference in podocyte numbers and the glomerular expression pattern of Par3 could be observed in adult *Scribble^Δpodocyte^* mice (Figure 5 E, Figure S2). During an one year follow up no difference in albumin to creatinine ratio could be detected between *Scribble^Δpodocyte^* and control mice (Figure 5 F), and *Scribble^Δpodocyte^* mice featured no increased susceptibility to glomerular stress in the BSA overload and the Adriamycin (ADR) model (Figure 5 G, H).

### Development of podocyte foot processes in cultured embryonic Scribble knockout kidneys and in circletail mutant mice

**Figure 6:**
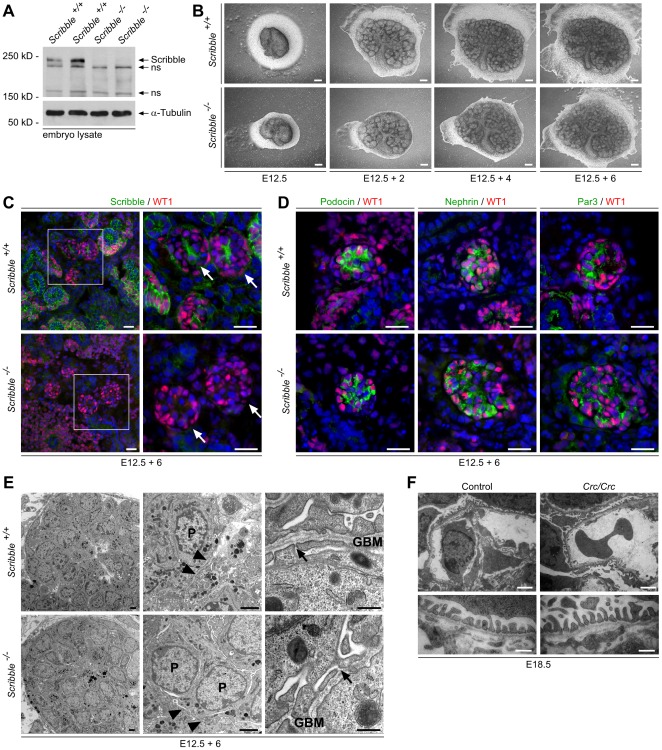
Development of podocyte foot processes in cultured embryonic *Scribble* knockout kidneys and in *circletail* mutant mice. (**A**) Western blot of total embryo lysates shows complete loss of Scribble protein in *Scribble* knockout embryos (*Scribble^−/−^*) (ns, non specific). (**B**) Kidneys of *Scribble* knockout and wildtype littermate embryos were harvested at P12.5 and grew in DMEM medium for 6 days. (**C**) Immunofluorescence staining against WT1, which is expressed in podocytes and embryonic kidney epithelial cells, and Scribble reveals complete loss of Scribble in *Scribble* knockout kidney culture (arrows indicate glomeruli), (**D**) while expression of the podocyte marker proteins Podocin and Nephrin as well as the apical polarity protein Par3 can be detected in *Scribble* knockout kidney culture glomeruli. (**E**) Electron micrographs show development of podocyte foot processes connected by slit diaphragms in *Scribble* knockout and wildtype kidney culture glomeruli (P, podocyte; GBM, glomerular basement membrane). Arrow heads indicate localization of foot processes, arrows indicate slit diaphragms. (**F**) *Circletail* mutant mice, bearing a mutation in the *Scribble* gene, which results in a shortened protein lacking third and fourth c-terminal PDZ domains, die during late embryonic development. Kidneys of *Crc/Crc* mutant mice and control littermates where harvested for electron microscopy at E18.5. Transmission electron micrographs display podocytes with normal foot process architecture and slit-diaphragms and without any obvious abnormalities. Scale bars: 400 µm in (B), 20 µm in (C) and (D), 2 µm in (E) left and middle panel, 500 nm in (E) right panel, 2 µm in (F) upper panel, and 500 nm in (F) lower panel.

Since *Cre* expression under control of the *NPHS2*-promoter starts late during glomerular development [24], [25], a developmental phenotype of *Scribble* knockout in podocytes might be masked in *Scribble^flox/flox^; NPHS2.Cre* mice. To study the effect of *Scribble* knockout on early glomerular development and podocyte differentiation, we analyzed kidneys of constitutive *Scribble* knockout mice. *Scribble^flox/flox^* mice were crossed with Cre deleter mice to create constitutive *Scribble* knockout mice (Figure 6 A). Most constitutive *Scribble* knockout animals die during embryonic development between E12.5 and E14.5, before podocyte maturation is finished. To monitor the glomerular development we harvested the kidneys at E12.5 for kidney culture experiments. Figure 6 B illustrates the development of *Scribble* wildtype and knockout kidneys in culture for 6 days. Immunofluorescence staining against WT1 and Scribble confirmed complete loss of Scribble in *Scribble* knockout kidney culture (Figure 6 C), while expression of the podocyte marker proteins Podocin and Nephrin as well as the polarity protein Par3 could be detected in glomeruli of *Scribble* knockout kidney cultures (Figure 6 D). Ultrastructural analysis disclosed podocyte foot processes connected by slit diaphragms in *Scribble* knockout and wildtype kidney culture glomeruli (Figure 6 E). We next analysed homozygous *circletail* mutant mice, which express a shortened Scribble protein lacking the third and fourth c-terminal PDZ domains leading to craniorachischisis, gastroschisis and late embryonic death around E18 [12]. No obvious morphological alteration of podocyte foot processes could be detected in these mice (Figure 6 F), indicating that Scribble is dispensable for regular foot process development.

## Discussion

In epithelial cells Par3 is located at the tight junctions marking the border between apical and basolateral membrane compartments [3]. Previously we demonstrated that Par3 co-localizes with the tight junction protein ZO-1 at the apical cell-cell contacts of immature podocytes as well as at the slit diaphragm of mature podocytes [2], [5]. Here we could demonstrate that another polarity protein, Scribble, is enriched in podocytes. During differentiation the immature cell-cell contacts and Par3 as well as Scribble translocate towards the basal aspects of the podocyte, followed by primary and foot process development (Figure 1). Interestingly, Scribble appears to localize basally of Par3 during podocyte differentiation as well as in mature podocytes. In agreement, in polarized MDCK cells it has been described that Scribble localizes basal of the tight junctions at the adherens junctions [9]. Strikingly, mature podocytes do not feature classical tight or adherens junctions but a unique cell-cell contact, the slit diaphragm, which bridges foot processes of neighbouring podocytes. The slit diaphragm displays characteristics of both, tight junctions with associated proteins such as ZO-1 [26], Jam4 [27] and Par3 [2] as well as adherens junctions with proteins like P-cadherin [28]. This unique cell-cell contact makes it difficult to predict the function of single junctional components. In MDCK epithelial cells knockdown of Scribble causes delayed tight junction assembly and disrupts cell adhesion [10]. However, it remained completely unclear whether Scribble also significantly contributes to the development and maintenance of podocyte slit diaphragms. Therefore, we generated podocyte-specific *Scribble* knockout mice. Unexpectedly, no morphological or clinical abnormalities could be detected in these mice (Figure 4 and 5). Since the *NPHS2*-promoter activates *Cre* expression relatively late during glomerular development [24], [25], a developmental phenotype of podocyte-specific deletion of *Scribble* might be masked. For this reason we analyzed constitutive *Scribble* knockout mice and *circletail* mice, in which Scribble is truncated after the second PDZ domain, which results in late embryonic death [12]. Constitutive *Scribble* knockout mice die intraembryonally between E12.5 and E14.5. To study glomerular development we performed kidney culture experiments. Podocytes in constitutive *Scribble* knockout kidney culture as well as podocytes of *circletail* mice develop foot processes being connected by the slit diaphragm showing no morphological abnormalities compared to control podocytes indicating that Scribble is dispensable for podocyte foot process development (Figure 6). However, if other basal polarity proteins are involved in podocyte shape formation, has to be addressed in future studies.

In summary, while Scribble seems to be important for general epithelial cell- [9] and pronephros [29] development, it appears to be dispensable for podocyte function and development. This unexpected result underlines the unique features of polarity programs shaping the complex three-dimensional podocyte architecture. Striking characteristics of developing podocytes are the expansion of apical membranes going along with a shrinking of basal membrane compartments (as illustrated in Figure 1 and 3). This appears to be congruent with the observation that podocyte differentiation is rather being driven by apical polarity complex signaling while Scribble signaling is not required for the specialized podocyte architecture.

## Materials and Methods

### Mice

LoxP sites were introduced into the introns 1 and 8 flanking *Scribble* exons 2–8 in 129S1/SvlmJ mouse embryonic stem cells, using a neo cassette as a selectable marker flanked by frt sites. Targeted stem cells were injected into blastocysts of C57BL/6J mice to obtain chimeric floxed mice. After germline transmission, the mice were crossed to C57BL/6J mice expressing flp recombinase to remove the neo cassette. Progeny containing a floxed *Scribble* allele lacking the neo cassette (*Scribble^flox/+^*) were further backcrossed to C57BL/6J mice. Cre-mediated recombination causes a frame shift and early stop of translation. In addition floxed mice were crossed to C57BL/6J Cre deleter mice to excise the loxP flanked genomic region (exons 2–8) and to generate heterozygous mice carrying the constitutive knockout allele (*Scribble^−/+^*) [30], [31]. Construction of the targeting vector, generation and injection of targeted stem cells and subsequent generation of chimeric mice, *Scribble^flox/+^* and *Scribble^−/+^* mice were performed by genOway (Lyon, France).

*NPHS2.Cre* mice were kindly provided by Lawrence Holzman (Renal, Electrolyte and Hypertension Division, University of Pennsylvania School of Medicine Philadelphia, PA, USA) [24]. *Scribble*-floxed mice (*Scribble^flox/flox^*) were crossed with *NPHS2.Cre* mice to generate podocyte-specific *Scribble* knockout mice *Scribble^flox/flox^; NPHS2.Cre* (*Scribble^Δpodocyte^*). *NPHS2.Cre* negative *Scribble^flox/+^* and *Scribble^flox/flox^* litter mates served as controls. Mice carrying the *circletail* allele of *Scribble* were purchased from The Jackson Laboratory.

For genotyping or detection of deletion of *Scribble* exons 2–8 DNA was isolated from tail clip or isolated glomeruli, respectively. For detection of WT or the loxP site, the primers were tccagttagcactcaggcgtcagg (forward) and cagctccgagaggttctcacagtcc (reverse). For detection of the deletion, primers were accccagtgctctctggtgtttttattg (forward) and cagctccgagaggttctcacagtcc (reverse). For detecting the *circletail* mutation, primers were ctagccctcccccccc (forward; locked nucleic acid primer) and cctgggactgagaaggacat (reverse). All animal studies were approved by the Committee on Research Animal Care, Regierungspräsidium Freiburg and by the Washington University Animal Studies Committee.

### Protein Overload and Subsequent Analysis

*Scribble^Δpodocyte^* mice and control littermates (n = 5 each) received endotoxin-free BSA (Sigma A9430) (250 mg/ml, dissolved in PBS) intraperitoneally for 4 consecutive days (10 mg/g body weight) [32], [33]. Urinary albumin excretion rates were analyzed before injections and at days 1 to 6 after the first injection.

### Adriamycin induced proteinuria

*Scribble^Δpodocyte^* mice and control littermates (n = 6 each) received one dose of intravenous Adriamycin (Sigma) in 0.9% NaCl (2 mg/ml) (15 µg/g body weight) [34]. Urinary albumin excretion rates were analyzed before injection and 2 and 5 weeks after injection.

### Urine and serum analyses

Urinary albumin and urinary creatinine were measured using a fluorimetric albumin test kit (Progen) and an enzymatic creatinine kit (Labor+Technik) following the manufacturer's instructions. Proteinuria was expressed as mg albumin/mg creatinine.

### Metanephric kidney culture

Timed matings were set up with constitutive *Scribble* heterozygous knockout mice; the date of the vaginal plug was designated as day 0. Metanephric kidneys were microdissected from the embryos at embryonic day 12.5 and cultured in Dulbecco's modified Eagle's medium containing 10% fetal calf serum and 1% Penicillin and Streptomycin at 37°C and 5% CO_2_ on 0.4 µm transwell inserts [35]. The medium was replaced every 48 h. The kidney cultures were harvested after up to 6 days in culture. For immunofluorescence staining, the cultures were fixed in 4% paraformaldehyde, dehydrated in 15% and 30% sucrose, frozen in OCT and sectioned into 6 µm thick sections.

### Morphological analysis

Kidneys were fixed in 4% paraformaldehyde, embedded in paraffin or Epon and further processed for PAS staining or transmission electron microscopy, respectively.

### Immunofluorescence staining of kidney sections

Kidneys were frozen in OCT compound and sectioned at 6 µm (Leica Kryostat). The sections were fixed with 4% paraformaldehyde, blocked in PBS containing 5% BSA and incubated for 1 hour with primary antibodies as indicated. After PBS rinse for several times, fluorophore-conjugated secondary antibodies (Invitrogen) were applied for 30 minutes. Images were taken using a Zeiss laser scan microscope equipped with a 63× water immersion objective or a Zeiss fluorescence microscope equipped with a 5×, a 20× and a 40× oil immersion objective. To determine the number of podocytes per glomerular section, kidney sections were stained against the podocyte nuclear marker WT1. WT1-positive cells were counted in 30 glomeruli per mouse per condition (n = 3 for each condition).

### Immunogold electron microscopy

Fixed samples of rat kidney were embedded in Lowicryl K4M resin (Electron Microscopy Sciences), and ultrathin sections were labeled by an indirect immunogold protocol, as described [36].

### Antibodies

Antibodies were obtained from Millipore (anti-Par3 rabbit pAb, 07-330, immunofluorescence dilution 1∶125), Progen (anti-Nephrin guinea pig pAb, GP-N2, immunofluorescence dilution 1∶100), Sigma (anti-α-Tubulin mouse mAb, T6199; anti-Podocin rabbit pAb, P0372, immunofluorescence dilution 1∶1200), Abcam (anti-WT1 rabbit pAb, ab15249, immunofluorescence dilution 1∶300) and Santa Cruz Biotechnology (anti-Scribble rabbit pAb, H-300, sc-28737, immunofluorescence dilution 1∶125; anti-Scribble goat pAb, C-20, sc-11049, immunofluorescence dilution 1∶100). Mouse monoclonal antibody against rat Podocalyxin was described before [37]. Nuclear staining reagent (To-Pro-3, T3605) and fluorophore conjugated secondary antibodies were obtained from Invitrogen.

### Statistical Analyses

Data were expressed as the mean ± SEM. Statistical comparisons were performed using two-tailed Student's t-test if not stated otherwise. Differences with P<0.05 were considered significant.

## Supporting Information

Figure S1 **Expression of Scribble in the developing and adult kidney.** Frozen kidney sections of newborn and adult Wistar rats were stained using antibodies against Scribble and the podocyte marker proteins (**A**) WT1 and (**B**) Nephrin. Scribble is expressed in glomeruli as well as in segments of the tubule system. Scale bars: 50 µm.
(TIF)

Figure S2 **Expression pattern of Par3 in glomeruli of** ***Scribble^Δpodocyte^*** **mice.** Frozen kidney sections of adult *Scribble^Δpodocyte^* and control mice were stained using antibodies against Par3 and the slit diaphragm protein Nephrin. No difference in the expression pattern of Par3 could be detected in *Scribble^Δpodocyte^* compared to control mice. Arrows indicate colocalisation of Par3 and Nephrin. Scale bars: 5 µm.
(TIF)
