# Correction: Role of the Polarity Protein Scribble for Podocyte Differentiation and Maintenance

**DOI:** 10.1371/annotation/2c0c1c61-0627-4794-b6f2-01fc4db84dcb

**Published:** 2014-01-03

**Authors:** Björn Hartleben, Eugen Widmeier, Nicola Wanner, Miriam Schmidts, Sung Tae Kim, Lisa Schneider, Britta Mayer, Dontscho Kerjaschki, Jeffrey H. Miner, Gerd Walz, Tobias B. Huber

A funding organization and grant to the third and last authors were incorrectly omitted from the Funding Statement. The following sentence should be read with the Funding Statement: "This study was supported in part by the Excellence Initiative of the German Research Foundation (GSC-4, Spemann Graduate School to NW and TBH)." 

