# Supplementary figures and images for: Role of the Polarity Protein Scribble for Podocyte Differentiation and Maintenance
Source: PLoS One. 2012 May 7;7(5):e36705. doi: 10.1371/journal.pone.0036705 (PMC3346764; doi:10.1371/journal.pone.0036705)

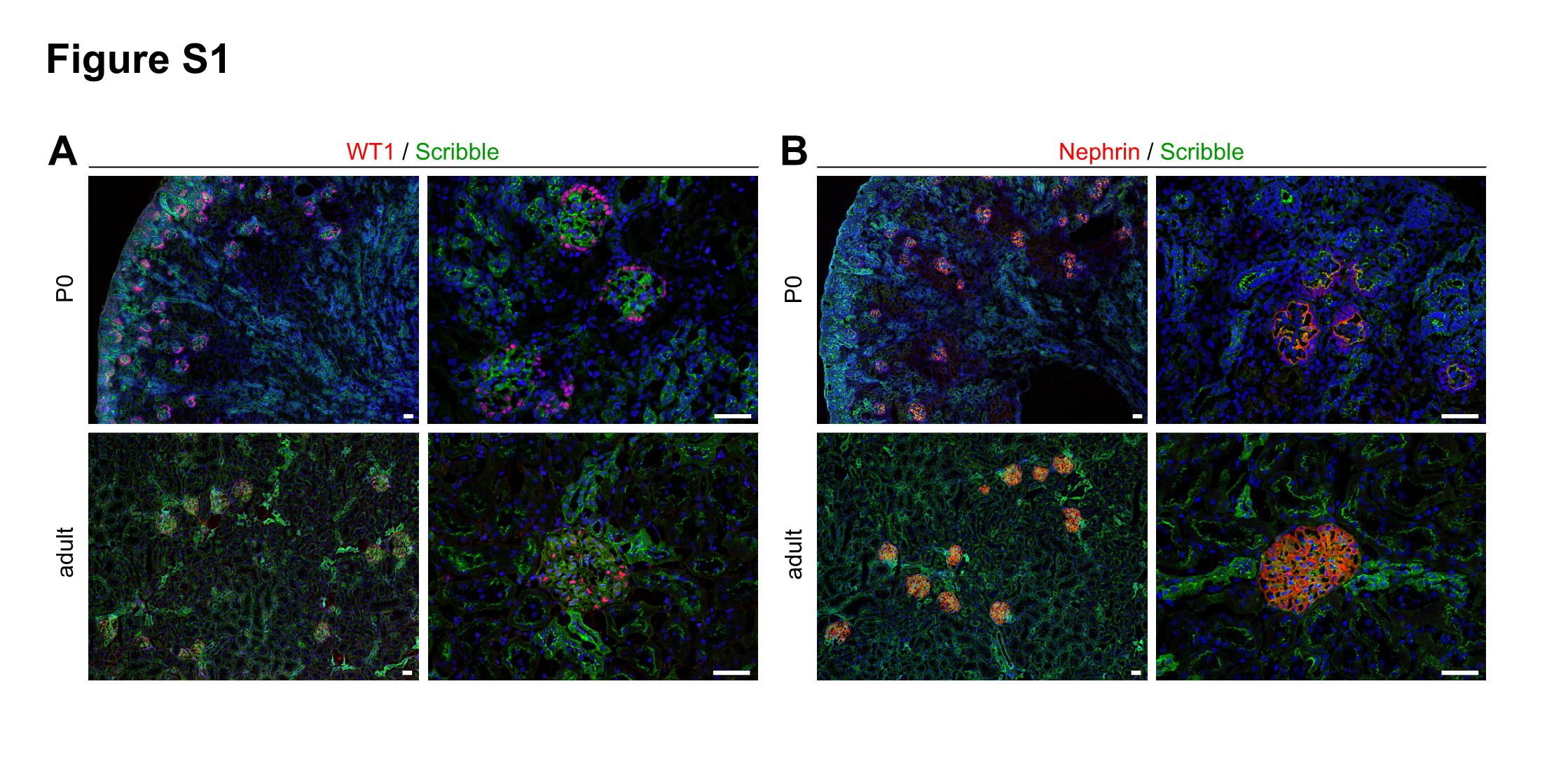

Supplement: Figure S1 — Expression of Scribble in the developing and adult kidney. Frozen kidney sections of newborn and adult Wistar rats were stained using antibodies against Scribble and the podocyte marker proteins (A) WT1 and (B) Nephrin. Scribble is expressed in glomeruli as well as in segments of the tubule system. Scale bars: 50 µm. (TIF) [file pone.0036705.s001.tif]

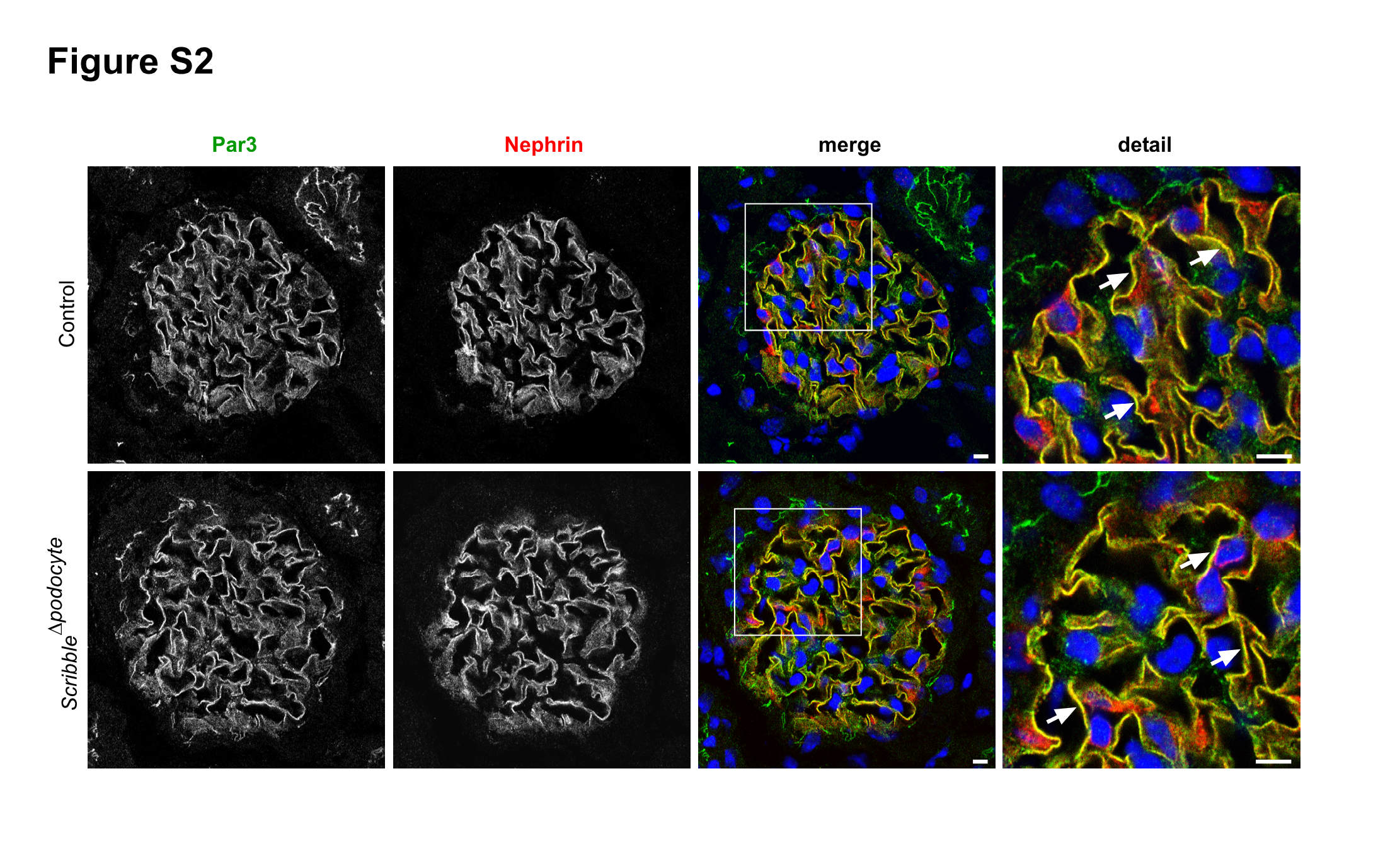

Supplement: Figure S2 — Expression pattern of Par3 in glomeruli of ScribbleΔpodocyte mice. Frozen kidney sections of adult ScribbleΔpodocyte and control mice were stained using antibodies against Par3 and the slit diaphragm protein Nephrin. No difference in the expression pattern of Par3 could be detected in ScribbleΔpodocyte compared to control mice. Arrows indicate colocalisation of Par3 and Nephrin. Scale bars: 5 µm. (TIF) [file pone.0036705.s002.tif]
